# Unitarianism

**Published:** 1860-03

**Authors:** 


### UNITARIANISM.

Do not be alarmed, reader! We are a doctor of physic, not of polemic theology. Besides, we don't believe in blazing away at the religious beliefs of any body, for who, worth converting, was ever converted by a doctrinal dispute? Our general observation is, that when a man leaves the church of his birth, and goes over to another, he either is not worth having, in a sense, or his new friends in course of time wish they had never seen or heard of him. To this there are exceptions.

The busiest of created beings is the old fellow down yonder. Not exactly "down" either, we rather think that to say "in and about us" would be more literally correct. To head him off, Christian people must be wide awake, and must sleep, if at all, with one eye open. The necessity for this vigilant look-out, is especially great in cities, where the personage in question manages so generally to form an alliance, offensive and defensive, with the editorial fraternity. With the exception of such papers as the *Courier and Inquirer* and the *Commercial Advertiser*, and perhaps one or two others, no man can tell any morning that he shall not sit down to his breakfast with an outraged moral or religious sentiment.

What a poor, unfortunate, commiserable individual is an old "bach." You can tell him a mile off, more or less. There is a peculiarity in his physiognomy that is unmistakable. Right nice old fellows some of them are, so friendly, so deferential to the ladies, willing to do almost any thing in the world for them "in reason!" One of them came navigating around to our office within a week. We had not seen him for years. He wanted a "boarding-place." He did not expect to live, merely to exist. He had been roughing it so long in the world, knowing nothing of the softening influences of wife, children, and home, he seemed to have no ambition, no anticipation of any thing softer than a board, hence he wanted our advice as to an eligible "boarding" place.

Now for the connection between the old boy, New-York editors, and this old "bach." The party first above named, as we said, is always very busy in "furthering his views," and being up to the ways of the world, he obtains the "assistance" of those who are most "handy" in aiding and abetting as to matters in hand. Thus he secures the editors first. So he procured several of them to advocate "Unitarianism," which they have done from time to time within a few months past, rather gingerly at first, so as to feel the public pulse, to break the ice like, throwing in an objurgatory now and then, so as not to excite special alarm all at once. One of these papers had gained our confidence somewhat, and through its representation we concluded to try Unitarianism the first convenient opportunity, which was "embraced" the moment the old "bach" gave out his idea. "Try the Unitarian plan awhile," said we.

"Unitarian! botheration on any thing unitarian. I hate every thing that has a unit about it. I have been a unit all my life, and I am becoming uniter every day. 'Friend after friend departs,' he and she, even now, I know almost nobody and nobody knows me, and very soon there will not be an eye to weep or a heart to sorrow, when this bag of bones is huddled into its last resting-place."

We saw that he was touchy, excitable, and fidgety, and at once put out a mollifying aura, and explained, that there were "unitary households" in New-York, where there were reported to be excellent rooms, accommodations, and company, at literally "cost" prices. This pleased him much, and he started out on a voyage of discovery. Now, our friend was an exemplary bachelor, a man of honor, of uprightness and morality. Meeting him on the street a few days later, we inquired "what progress?"

"All very fine. Large house. Prices satisfactory. Splendid parlors, faultless mirrors, curtains of the richest kind, and carpets of the orient,

"Soft as downy pillows are."

"Are you beatifically ensconced therein? Are you delightfully domiciled in such an elysium?"

"My dear Doctor, not a bit of it. I'm not quite green enough for that."

"What's the matter?"

"I don't like the principles of the place."

"Principles? Why they hav'nt got but one. One principle and one inference. They believe that 'cost' should be the 'limit of price,' and therefore, the millennium is at hand, as heretofore every thing has 'cost more than it came to,' hence all the hard times and misery in the world."

"Now, Doctor! you have known me at home and abroad, for very near a quarter of a century. We have slept under the same tree. We have bedded together on the same blanket on the boundless prairie, in forests untrodden by white man's foot before. We have watched at midnight against the common enemy on the same shipwrecked shore. We have divided together the last half-pint of water, and through it all, you have found me a man, and woman's best friend. The principal said to me: Our 'society' is unexceptionable. We estimate people from what we see. It is not our business to inquire if any woman is wife, maid, or widow. And now, Doctor, not to misrepresent, for I would not do a solitary human soul the smallest mite of harm, so I will not pretend to repeat the precise words, for my memory is poor, and I may not have heard very well, or the gentleman may have expressed himself imperfectly or awkwardly; all I can say is, that the impression made upon my mind, from what I thought I heard him say was, that he never inquired of ladies coming there whether they were maid, wife, or widow, and when they went away, they were all the same. Now Doctor, the good book says of the old, that 'fears are in the way.' I am getting old, and hence must be getting fearful. There is so much iniquity in this world, that I have become a boy again, and think there is, or may be, a bug-a-boo behind every stump; and may be, my inferences were illegitimate, deriving their impression from the auras of the homœopathic principles and entities about me. I am dull too. The brightness of youth is past. My perspicacity is more or less obtunded. The problem is beyond my solution. I believe I will stop in at Prof. Mitchell's on Union Square, only a block away. I understand he can solve problems a million, billion, quadrillions of miles away, and make them as plain as if you had them in your hand. The problem which I wanted him to resolve into its original elements, and to expound with that crystal clearness for which he is so famous, is simply this,· If a lady becomes a Unitarian, whether the blooming sweet 'queecher' of enrapturing seventeen, or the captivating widow of twenty-five, or of the angel wife, pure, loving, and true—I say, what I want to know is this, how they all come away the same when they get tired of the place. And if when they become the same, they naturally act like the people who hold the doctrine of falling from grace, they turn in and then turn out, and 'try the spirits,' first of one household and then another, getting to believe in the doctrine of Cowper, that

'Variety 's the spice of life.' "

We saw at once that our old-time friend was in a fog, just as dense as the one by reason of which our ship went to the bottom, leaving us "high and"—wet as drowned rats, to swim for shore or go right down to Davy Jones' locker. Once again we threw out the mollifying aura, quieted his nerves, and calmed his apprehensions, by saying he had better take things by the smooth handle, and cultivate the amiable spirit of him who "thinketh no ill" of his neighbor, advising him as we parted on the sidewalk, for we had been standing "stock still" on the same spot all this time, at the imminent risk of having our eyes gouged out by the expanded umbrellas of careless pedestrians, that it was an old-time policy of ours that the safest plan was the best plan in morals as well as in medicine; and further, that it was a prime principle in "doctoring," which common-sense corroborated, that if a man was made sick by any particular thing once, he should simply avoid it ever after, and that although very few persons had sense enough to adopt that rule, we thought there would be no difficulty in his applying the principle of the thing to the case in hand, to wit: As it was perfectly certain that being a unit had injured him, he had better give all unities a wide berth for the remainder of his days.

"Why Doctor! you are eloquent in this drizzle; cold water don't squench your oratory. You have convinced me, sir. I will take you hereafter, not only as my doctor, but as my priest. I'll pin my faith to the very tipmost extremities of your trowserloons. I despise unity in any shape, matter, or form; it is my inmost antipathy. My stars! what handsome young lady was that who passed just now? I'll step up and lend her my 'umbrell.' Oh! that's cousin Loo, she is over at the 'Everett,' the handsomest woman in New-York, a poetess too, rich, a wife and mother." His eyes fell, and he walked slowly and sadly away, muttering, "Every body double and happy but me," ending in a strain of song

"I won't be an un."

We heard no more. We are huge on being strictly literal, and suppose that if we had heard the remainder of the sentence it would have run thus:

"I won't be an unitarian."

Reader, may be he is, like you, wise too late!

